# Delayed-Onset Malignant Hyperthermia With Disseminated Intravascular Coagulation Following Successful Prior Anesthetic Use: A Case Report

**DOI:** 10.7759/cureus.117261

**Published:** 2026-09-30

**Authors:** Nicole Buleza, Leena Ramadan

**Affiliations:** 1 Internal Medicine, Penn State Health, Reading, USA; 2 Internal Medicine, Inspira Health Network, Mullica Hill, USA; 3 Critical Care Medicine, Cooper University Hospital, Camden, USA

**Keywords:** critical care and internal medicine education, critical care anesthesiology, disseminated intravascular coagulation (dic), malignant hyperthermia (mh), risk of malignant hyperthermia

## Abstract

Malignant hyperthermia (MH) is a serious perioperative reaction associated with exposure to volatile inhalational anesthetics and/or succinylcholine. Severe episodes may lead to widespread physiologic instability, including coagulopathic complications. This case describes an otherwise healthy middle-aged man who developed MH complicated by disseminated intravascular coagulation (DIC) during a subsequent surgery for a left ankle fracture. MH reactions can be unpredictable due to the involved genes' incomplete penetrance, which is why MH does not always occur each time an MH-susceptible patient is exposed to a triggering agent. This case highlights the importance of maintaining vigilance for MH even in patients with prior uneventful anesthetic exposures. Patients with suspected MH reactions should be referred for genetic testing, with important implications for future anesthetic management of both the patient and their family members.

## Introduction

Malignant hyperthermia (MH) is a rare, life-threatening hypermetabolic crisis triggered by exposure to volatile anesthetics or succinylcholine. MH reactions occur most frequently in males, with the highest incidence occurring around 18 years of age, but have occurred as late as 78 years of age. MH reactions occur infrequently relative to the total number of anesthetic exposures, with the incidence of MH episodes ranging from 1:10,000 to 1:250,000 anesthetics [1]. However, the prevalence of genetic abnormalities associated with MH susceptibility, such as ryanodine receptor 1 (RYR1) and calcium voltage-gated channel subunit alpha 1S (CACNA1S), among others, is estimated to be as high as one in 400 individuals. In severe cases, MH can progress to disseminated intravascular coagulation (DIC), where excessive clot formation depletes clotting factors, leading to simultaneous bleeding and thrombosis [1]. The following describes a case of MH complicated by DIC in an otherwise healthy 57-year-old male during a subsequent surgery for a left ankle fracture.

## Case presentation

A 57-year-old male with no known significant medical history presented following a trimalleolar left ankle fracture during a skiing trip. He denied any prior issues with local anesthesia as well as any family members with known anesthetic complications. The patient was deemed an American Society of Anesthesiologists (ASA) Class I physical status, and he underwent open reduction and internal fixation of his left tibia/fibula with general anesthesia. During the case, the patient received sevoflurane as the induction and maintenance anesthetic agent, with the total time under anesthesia being approximately 60 minutes. The initial surgery had no complications.

A week later, the patient underwent a second surgery to remove the external fixator. Again, sevoflurane was used for induction and maintenance, with the total time under anesthesia being slightly over 90 minutes. However, as the patient was being sutured closed, the patient became hypotensive, febrile, diaphoretic, and hypercapnic with end-tidal carbon dioxide (ETCO_2_) > 100, which were concerning for MH. The MH hotline was immediately called, and he was started on intravenous fluids, a 2.5 mg/kg bolus of dantrolene, norepinephrine, and transferred to the intensive care unit, where an arterial line and axillary ice packs were placed. Upon arrival to the intensive care unit, he was still febrile to 38.7°C and shortly after began bleeding from his nose and arterial line. Lab work demonstrated low fibrinogen level, elevated coagulation factors and D-dimer, with an International Society of Thrombosis and Haemostasis (ISTH) score of 6, consistent with overt DIC. Additionally, he was mildly hyperkalemic, with further labs consistent with moderate rhabdomyolysis, transaminitis, and severe respiratory and metabolic acidosis. Urine myoglobin level returned severely elevated (Tables 1-3).

**Table 1:** Complete blood count, basic metabolic panel, and arterial blood gas during hospitalization. WBC: white blood cell; Hgb: hemoglobin; Plt: platelets; CO_2_: carbon dioxide; PaCO_2_: partial pressure of arterial carbon dioxide; PaO_2_: partial pressure of arterial oxygen; HCO_3_: bicarbonate

	Prior to Surgery	During Surgery	Day 1 Post-ICU Admission	Day 2	Day 3	Day 5	Day 8	Reference Range
WBC	10.9	-	11.0	12.5	9.5	8.2	8.3	4-11 x 10^3^/µL
Hgb	12.0	-	8.8	8.0	8.2	8.3	8.7	12-17 g/dL
Plt	247	-	89	88	100	152	286	140-380 x 10^3^/µL
Creatinine	-	-	1.92	2.13	2.52	1.16	-	0.7-1.3 mg/dL
Sodium	-	-	134	138	141	141	-	136-145 mmol/L
Potassium	-	-	5.4	4.2	3.5	3.6	-	3.5-5.1 mmol/L
CO_2_	-	-	18	21	26	24	-	22.0-30.0 mmol/L
pH	-	6.857	7.246	7.378	7.4	-	-	7.35-7.45
PaCO_2_	-	150	42	31.4	35	-	-	35.0-45.0 mmHg
PaO_2_	-	99.6	260	184	122	-	-	74.0-108.0 mmHg
HCO_3_	-	14.1	18.3	18.5	21.8	-	-	21.0-29.0 mmol/L

**Table 2:** Coagulability workup and liver function testing. PT: prothrombin time; PTT: partial thromboplastin time; INR: international normalized ratio; ALT: alanine aminotransferase; AST: aspartate aminotransferase; FEU: fibrinogen-equivalent units

	Day 1 Post-ICU Admission	Day 2	Day 3	Day 4	Day 8	Reference Range
PT	17.5	22.5	23.7	21.8	-	9.7-13.3 seconds
PTT	62.3	35.2	25.6	26.8	-	22.2-37.6 seconds
INR	1.6	2.3	2.2	1.9	-	0.8-1.2
D-dimer	>128,000	>128,000	>128,000	122,648	-	<500 ng/mL FEU
Fibrinogen level	<80	<80	136	184	-	169-444 mg/dL
Triglycerides	72	-	-	-	-	<150 mg/dL
Alkaline phosphatase	86	86	83	119	126	46-116 U/L
ALT	271	1744	2206	1898	931	10-49 U/L
AST	399	1774	3331	1326	251	0-34 U/L
Total bilirubin	1.2	1.8	2.3	1.9	1.3	0.3-1.2 mg/dL
Direct bilirubin	-	1.1	1.3	1.3	0.7	0-0.3 mg/dL

**Table 3:** Other ancillary testing. CK: creatine kinase

	Day 1 Post-ICU Admission	Day 2	Day 3	Day 4	Day 5	Reference Range
CK	12,307	12,225	10,068	5,202	1,501	46-171 U/L
Myoglobin urine level	-	2,660	-	-	-	0-24 µg/L

Blood cultures were obtained, and intravenous lactated Ringer’s solution was initiated at 200 mL/h, along with vancomycin and cefepime. He received a total of 15 units of cryoprecipitate to raise fibrinogen levels above 150, several maintenance doses of dantrolene 1 mg/kg every six hours, as well as norepinephrine and three units of fresh frozen plasma (FFP) over the course of the next two to three days. The patient was successfully extubated on the evening of day 3 and eventually was discharged to subacute rehabilitation.

## Discussion

MH was the most consistent explanation for this patient’s intraoperative crisis, and other hypermetabolic syndromes were effectively excluded based on clinical context and the perioperative findings. The patient was not taking any regular medications, which substantially reduces the likelihood of neuroleptic malignant syndrome or serotonin syndrome, both of which require exposure to specific pharmacologic triggers. The hemodynamic profile was also inconsistent with pheochromocytoma; although pheochromocytoma can produce elevated end‑tidal CO_2_, it typically presents with marked hypertension or hypertensive crisis rather than hypotension as observed in this patient. Thyroid storm was similarly unlikely, as patients generally exhibit progressive symptoms (weight loss, tachyarrhythmias, agitation) in the days preceding decompensation. This patient was entirely asymptomatic prior to surgery; however, while a thyroid panel was not obtained, his presentation lacked the typical prodrome that would be expected. Taken together, the absence of medication triggers, the incompatible hemodynamic pattern, and the lack of prodromal endocrine symptoms support MH as the most likely etiology. Additionally, the Larach Clinical Grading Scale evaluates clinical signs and laboratory findings across several physiological categories to rank the likelihood of MH. This patient had a score of 43, placing him in the very likely range for a MH event.

MH is a very real threat to patient safety, even in patients with prior uncomplicated anesthetic histories. The most implicated genetic mutation is the RYR1 gene, which is inherited in an autosomal dominant pattern; however, it has incomplete penetrance, meaning individuals with the altered gene may not always experience the condition, which is why MH does not always occur each time an MH-susceptible patient is exposed to a triggering agent [1,2]. MH should be immediately recognized in any patient presenting with tachycardia, unexplained rise in ETCO_2_, hyperkalemia, muscle rigidity, or hyperthermia after exposure to anesthetics [1,3]. Hyperthermia is often a late sign and may not be present at the time of diagnosis.

The pathophysiology involves a susceptible individual being exposed to anesthetics or succinylcholine, which causes abnormal activation of the RYR1 channel. Notably, mutations in the CACNA1S and STAC3 genes further upstream of RYR1 have also been implicated in MH but to a lesser degree. Regardless, the result is uncontrolled calcium release, as the mutated RYR1 channel becomes hypersensitive, causing excessive calcium release from the sarcoplasmic reticulum into the muscle cell’s cytoplasm. This increased calcium leads to sustained muscle contraction, resulting in sinus tachycardia and also leads to increased CO_2_, resulting in hypercapnia [1,4,5]. If untreated, the rapid depletion of adenosine triphosphate (ATP) from the muscle contractions leads to rhabdomyolysis, resulting in elevated creatine kinase (CK) levels (often exceeding 20,000 units/L in the first 12-24 hours), hyperkalemia, and renal failure. DIC is recognized as one of the most serious complications documented in untreated or severe MH [3,4,6]. As the hypermetabolic crisis worsens, hyperthermia leads to endothelial damage in combination with muscle breakdown from rhabdomyolysis and proinflammatory states, leading to excessive thrombin generation. As microthrombi form throughout the vasculature, they consume clotting factors and platelets, resulting in the manifestations of DIC such as epistaxis or oozing around the arterial line, as seen in our patient, as well as his low fibrinogen level and elevated coagulation factors. The likelihood of any complication (DIC, rhabdomyolysis, renal failure, etc.) increases 2.9 times for every 2°C increase in body temperature and 1.6 times for each 30-minute delay in dantrolene administration [4].

Treating promptly with dantrolene, cooling, and vasopressor support as needed are vital to patient outcomes. Dantrolene sodium is a specific antagonist that inhibits the release of calcium from the sarcoplasmic reticulum and should be administered by rapid intravenous pushes beginning at a minimum loading dose of 2.5 mg/kg, repeated until symptoms subside or the maximum dose of 10 mg/kg has been reached. Treatment should be continuous until symptoms subside, as the effective dose depends on the individual's degree of susceptibility, length of exposure to the triggering agent, and time elapsed between onset and treatment initiation. Post-crisis follow-up with 4 to 8 mg/kg/day of oral dantrolene in divided doses should be administered for one to three days following a crisis to prevent recurrence [7].

These patients’ electrocardiograms should be closely monitored, and labs including DIC panel, arterial blood gas, complete blood count (CBC), basic metabolic panel (BMP), CK, and liver function tests should be ordered. Sufficient fluids, vasopressors, dantrolene, and plasma/blood products should be readily available, as anticipating complications of MH, such as DIC, and having the necessary products available can save lives.

## Conclusions

This case highlights the importance of situational awareness for MH even in patients with prior uneventful anesthetic exposures. The unpredictable nature of MH susceptibility, combined with the potential for life-threatening complications such as DIC, underscores the need for immediate recognition and aggressive treatment. Early identification of hypermetabolic signs, particularly unexplained hypercapnia, followed by prompt administration of dantrolene and supportive therapies such as correction of coagulopathies, is essential to patient outcomes. Patients with suspected MH reactions should be referred for genetic testing, with important implications for future anesthetic management of both the patient and their family members. This patient's successful recovery demonstrates that even severe MH crises complicated by DIC can be managed effectively with rapid recognition and intervention.
